# In This Issue

**Published:** 1995

## A PRIMER ON IMAGING

During the past 30 years, increasingly sophisticated imaging techniques have been developed that enable researchers and clinicians to examine both the structure and function of living tissue. This introductory article by John J. Doria provides background information on the imaging technology in use today and reviews how these techniques may be applicable to alcohol research. Structural imaging techniques, such as computed tomography (CT) and magnetic resonance imaging (MRI) are used to generate computer images of living tissue. Functional imaging techniques, such as positron emission tomography (PET) and magnetic resonance spectroscopy (MRS), permit scientists to investigate cell activity by tracking blood flow and energy metabolism. Electrical and magnetic energy resulting from cell activity also can be detected using sensitive imaging techniques, such as electroencephalogy (EEG) and magnetoencephalography (MEG). Each of these sophisticated imaging tools holds promise for the field of alcohol research. (pp. 261–265)

## STRUCTURAL BRAIN ALTERATIONS ASSOCIATED WITH ALCOHOLISM

Pathological studies were the first to demonstrate a link between chronic, heavy alcohol consumption and structural changes in the brain. It was unknown, however, whether the changes seen in these postmortem studies were truly representative of the alterations that occur in living tissue. With the advent of imaging techniques such as CT and MRI, researchers were able to confirm that the brain alterations first described in pathological studies are present in living tissue. Margaret J. Rosenbloom and Drs. Adolf Pfefferbaum and Edith V. Sullivan review findings obtained with MRI which suggest that the volume of brain tissue decreases over time in chronic heavy drinkers. The authors also report that recovery of tissue volume was observed in the subjects after a period of abstinence. The authors note that structural changes in the brain have yet to be correlated to specific cognitive deficits described in alcoholics. (pp. 266–272)

## PRENATAL EXPOSURE TO ALCOHOL: WHAT THE IMAGES REVEAL

Alcohol’s devastating effects on the unborn fetus have been documented since the 1970’s. Such alcohol exposure causes abnormalities in a child’s physical as well as psychological well-being. As noted by Drs. Sarah N. Mattson and Edward P. Riley, the brain appears to be especially sensitive to prenatal alcohol exposure, the effects of which are permanent. The authors discuss how MRI is being used to document patterns of alterations in the brain associated with prenatal exposure to alcohol. In addition, they discuss how such information may ultimately help treatment professionals better define ways to help these children overcome cognitive deficits resulting from early exposure to alcohol. (pp. 273–278)

## IMAGING STUDIES OF AGING, NEURODEGENERATIVE DISEASE, AND ALCOHOLISM

Brain imaging techniques allow researchers and clinicians to study and diagnose different causes of dementia, such as neurodegenerative diseases (e.g., Alzheimer’s disease), alcoholism, and the normal aging process. As noted by Drs. Jamie L. Eberling and William J. Jagust, imaging technology can detect shrinkage of brain tissue or disturbances in brain metabolism associated with different types of dementia. Although most forms of dementia have similar clinical characteristics, subtle differences in brain physiology also may exist. Based on these differences, clinicians can better diagnose a patient’s condition and more accurately determine the appropriate treatment and prognosis. (pp. 279–286)

## IMAGING OF THE HEART: POTENTIAL APPLICATION TO ALCOHOL-INDUCED HEART DISEASE

More than 200,000 Americans suffer from alcohol-induced heart disease. One type of heart disease, alcoholic cardiomyopathy, directly affects the heart muscle and is associated with chronic alcohol use. Alcoholic cardiomyopathy develops through a variety of mechanisms, including disruption of the heart muscle’s metabolism and energy production. Several imaging techniques are available to aid clinicians in detecting the structural and functional changes in the heart muscle brought on by alcohol. Dr. Steven R. Bergmann reviews some of these techniques and presents recent findings on alcohol’s effects on the heart and its metabolism. (pp. 287–292)

## MONITORING THE BRAIN’S RESPONSE TO ALCOHOL WITH POSITRON EMISSION TOMOGRAPHY

Measurements of blood flow and energy metabolism in the brain are sensitive indicators of brain function. Drs. Nora Volkow and Gene-Jack Wang and Mr. John J. Doria describe how PET is used to obtain these measurements. Such information helps to identify specific brain regions affected by alcohol and to investigate mechanisms of alcohol-induced cognitive and behavioral impairment. PET studies in alcoholics have shown that alcohol withdrawal alters metabolism in certain regions of the brain, even in the absence of detectable neurological or psychological cognitive functions. The authors cite recent data on PET as they explore a possible mechanism for some of alcohol’s effects and provide evidence to support the heritability of alcoholism. (pp. 296–299)

## VISUALIZING NEURAL PATHWAYS AFFECTED BY ALCOHOL IN ANIMALS

Alcohol and other drugs modify the activity of certain groups of nerve cells, or neural pathways, in the brain. Research is under way in animals to discover if all drugs act on a common neural pathway and to determine if long-term exposure to alcohol affects different pathways than short-term exposure. Drs. David Lyons, Linda J. Porrino, and Susanne Hiller-Sturmhöfel provide an overview of several imaging techniques used by scientists to address these questions. The authors also review studies using these imaging techniques to demonstrate that the specific neural pathways affected by alcohol depend on the amount of alcohol consumed, the time elapsed since ingestion, and the animal’s history of alcohol exposure. (pp. 300–305)

## MAGNETIC RESONANCE SPECTROSCOPY OF THE BRAIN IN ALCOHOL ABUSE

After ingestion, alcohol rapidly enters the bloodstream, then easily crosses into the brain, where it produces most of its effects on behavior. Until recently, the ways in which alcohol affects brain function were not fully understood. The development of more sophisticated imaging techniques makes it possible for researchers to peer inside the living human brain without invasive surgery to reveal the subtle interactions taking place between brain molecules. Drs. George Fein, Dieter J. Meyerhoff, and Michael W. Weiner describe research using MRI and MRS. This research suggests that alcohol can bind to cell membranes in the brain and that this interaction changes when people develop a tolerance to alcohol’s effects. Such research may help clarify how alcohol produces effects on the brain as well as why people differ in their response to alcohol and their vulnerability to alcoholism. (pp. 306–314)

## MEASURING ELECTRICAL ACTIVITY OF THE BRAIN: ERP MAPPING IN ALCOHOL RESEARCH

The recording of brain electrical activity from scalp electrodes provides sensitive measures of brain function as it occurs. Event-related potentials (ERP’s), which record the subject’s response to a sensory stimulus, are extremely sensitive to acute and chronic effects of alcohol. David B. Chorlian and Drs. Bernice Porjesz and Howard L. Cohen describe how computerized mapping can graphically depict ERP data to determine the influence of alcohol on ERP generation in specific brain regions. These data are helpful in investigating alcohol’s effects on information processing as well as the genetic component of vulnerability to developing alcoholism. (pp. 315–320)

